# Therapeutic Approaches for HTLV-1-Associated Myelopathy/Tropical Spastic Paraparesis: Current and Emerging Strategies

**DOI:** 10.3390/pathogens15050555

**Published:** 2026-05-20

**Authors:** Tatsufumi Nakamura, Katsuya Satoh

**Affiliations:** 1 Division of Neurology, Yohkoh Rehabilitation Hospital, Hakujyujikai, 855-1 Yamatecho, Sasebo 857-0022, Japan; 2 Unit of Medical and Dental Sciences, Department of Health Sciences, Nagasaki University Graduate School of Biomedical Sciences, 1-7-1 Sakamoto, Nagasaki 852-8501, Japan; satoh-prion@nagasaki-u.ac.jp

**Keywords:** HTLV-1, HAM/TSP, treatment, anti-HTLV-1 effect, immunomodulation

## Abstract

Human T-cell leukemia virus-1 (HTLV-1)-associated myelopathy/tropical spastic paraparesis (HAM/TSP) is a chronic inflammatory disease of the spinal cord induced by immunological activation due to high HTLV-1 proviral load in the peripheral blood. HAM/TSP is representative of HTLV-1-related inflammatory diseases, and its main neurological symptoms—namely, motor dysfunction of the lower extremities through spastic paraparesis with urinary disturbance—are progressive and lead to deterioration in the quality of life of patients once these dysfunctions develop. Therefore, novel and safe therapeutic regimens are needed, enabling patients to commence treatment as soon as possible after the diagnosis of HAM/TSP. To date, various treatments have been developed for the correction of the associated immunological or virological abnormalities, which have produced some good results. However, there are still many problems, such as insufficient treatment effects and side effects. In addition, most of these treatments have only been characterized in the short term, being in the open trial phase, and it remains unclear whether or not they are suitable for the long-term treatment of HAM/TSP induced by a chronic inflammatory status. Thus, we need effective therapeutic regimens with safety for long-term or even lifelong courses of treatment. In this review, we summarize the clinical trials conducted to date for various therapeutic approaches, including representative regimens against HAM/TSP, while touching on the problematic issues. In addition, we discuss several agents with the potential to enable the development of novel therapeutic regimens as emerging interventions for further investigation in future research.

## 1. Introduction

**Figure 1:**
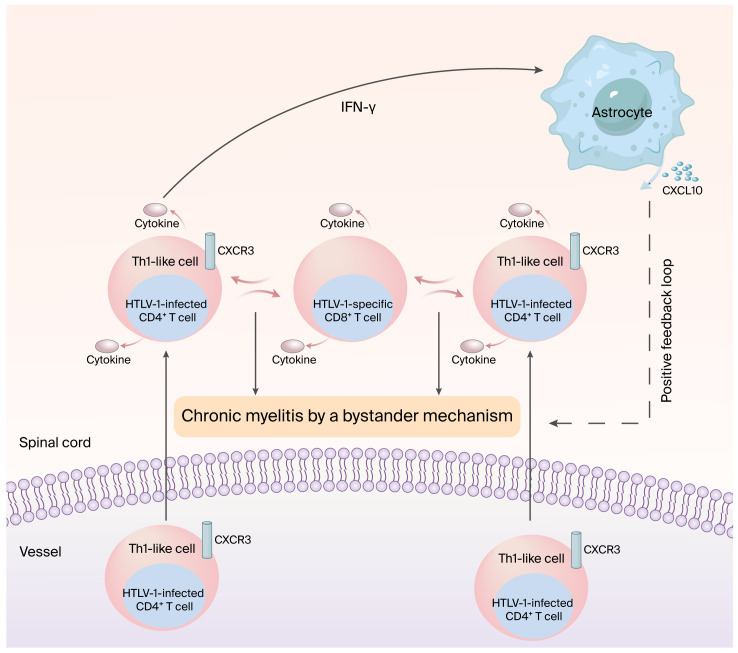
The proposed mechanism for the development of HAM/TSP through an inflammatory cascade. In the induction of chronic myelitis, a bystander mechanism—such as the destruction of surrounding tissues by the interaction between infiltrated Th1-like HTLV-1-infected CD4^+^ T cells and HTLV-1-specific CD8^+^ cytotoxic T cells (CTLs)—plays a critical role. For chronic myelitis developed via a bystander mechanism, a positive feedback loop formed through the expression of chemokine CXCL10 (a ligand of CXCR3) by astrocytes via interferon-γ (IFN-γ) from infiltrated Th1-like HTLV-1-infected CD4^+^ T cells might be involved in the maintenance and promotion of chronic myelitis.

Human T-cell leukemia virus-1 (HTLV-1)-associated myelopathy/tropical spastic paraparesis (HAM/TSP) is a chronic progressive myelopathy characterized by spastic paraparesis induced by bilateral pyramidal tract involvement, with urinary disturbance [1,2]. Although HTLV-1 is a powerful causative agent of inflammation [3,4], the chronic inflammation of the spinal cord—mainly the lower thoracic cord—under high HTLV-1 proviral load in peripheral blood mononuclear cells (PBMCs) is involved in the process of chronic progressive myelopathy [5,6]. However, the exact mechanisms by which HTLV-1 infection induces chronic inflammation of the spinal cord are still unclear. In this context, a long-standing bystander mechanism—such as the destruction of surrounding tissues under the interaction between Th1-like HTLV-1-infected CD4^+^ T cells and HTLV-1-specific CD8^+^ cytotoxic T cells (CTLs) in the spinal cord—has been proposed as a conceivable hypothesis for the development of HAM/TSP, as depicted in Figure 1 [7]. A recent report demonstrated that a positive feedback loop forms through the expression of chemokine CXCL10 (a ligand of CXCR3) by astrocytes via interferon-γ (IFN-γ) from infiltrated Th1-like HTLV-1-infected CD4^+^ T cells, which may be involved in the maintenance and promotion of chronic myelitis through a bystander mechanism [8] (Figure 1). In addition, non-HTLV-1-infected CXCR3^+^ activated cells are attracted by CXCL10, which might have an additive effect that drives this positive feedback loop. Thus, a long-standing bystander mechanism leading to an inflammatory cascade plays an important role in the pathological process occurring in the spinal cord of HAM/TSP patients.

Since the discovery of HAM/TSP, numerous abnormal findings from both immunological and virological perspectives have been reported [5,6,9]. Therefore, based on the concept that these abnormalities are involved in the development of chronic inflammation in the spinal cord through an inflammatory cascade, many therapeutic approaches have been developed for the correction of these abnormalities. Indeed, various treatments for HAM/TSP patients have been introduced to date [10]. Although an ideal therapeutic strategy against HAM/TSP has still not been established for various reasons, including insufficient treatment effect and side effects, the available strategies have still yielded valuable results. Therefore, for the development of a new therapeutic strategy, we need to reconsider the outcomes (including side effects) associated with existing treatments.

Considering strategies for the treatment of HAM/TSP to intercept the inflammatory cascade, they are categorized into two groups—treatments focusing on (1) anti-viral effects, leading to the correction of inflammatory conditions, and (2) anti-inflammatory effects via immunomodulation. The various treatments reported to date are presented in Table 1. Almost all of them have undergone therapeutic trials for small numbers of HAM/TSP patients in the short term as open-label trials. Therefore, in this review, we discuss the therapeutic trials performed to date for different therapeutic approaches, considering representative regimens against HAM/TSP, while also introducing recent reports on the efficacy of new regimens and discussing their potential as candidates for new therapeutic strategies.

**The search methodology**

We performed a targeted search of PubMed until March, 2026. The search strategy included the following keywords: HTLV-1, HAM, HAM/TSP, and Treatment. The search was limited to articles published in the English language. The inclusion criteria were as follows: (1) peer-reviewed articles; (2) human studies. The exclusion criteria were as follows: (1) conference abstracts; (2) editorials. Priority was given to studies with a rigorous methodology, including systematic reviews and mechanistic research. We included additional key papers identified by expert selection.

(1) **The therapies focusing on anti-viral effects** (Table 1)

The development of HAM/TSP is based on long-standing chronic inflammation in the spinal cord as mentioned above. However, as the HTLV-1-infected CD4^+^ T cells inducing this situation are the first responders in the development of HAM/TSP, the elimination of HTLV-1-infected cells from the peripheral blood is considered a reasonable therapeutic strategy against HAM/TSP. Various treatments have been developed for this purpose.

(a) **Reverse transcriptase inhibition**

Some nucleoside analogs have been shown to block HTLV-1 replication through inhibition of reverse transcriptase (RT). Previously, certain benefits of treatment with the RT inhibitors zidovudine or lamivudine have been reported [11,12]. However, this efficacy was contradicted when assessing combination therapy with zidovudine and lamivudine in a randomized, double-blind, placebo-controlled study as a regimen targeting HTLV-1 as a treatment for HAM/TSP [13]. The clinical effects, including motor disability score, gait, and bladder function, as well as changes in laboratory markers in the peripheral blood, including HTLV-1 proviral load and T cell subpopulation, were compared between groups treated with combined therapy or placebo therapy, and no significant differences were observed between the two arms. This finding strongly suggests that neither RT inhibitor can reduce the HTLV-1 proviral load, at least in an in vivo setting, in HAM/TSP patients. The fact that increased proliferation of HTLV-1-infected cells—rather than new infection through cell-to-cell spread—plays an important role in the maintenance of a high HTLV-1 proviral load in the peripheral blood of HAM/TSP patients [14] may explain the inefficacy of treatment with RT inhibitors.

(b) **Integrase inhibition**

The efficacy of raltegravir—an integrase inhibitor used as a therapeutic drug in patients with human immunodeficiency virus 1—for treatment of HAM/TSP has recently been reported [15]. Treatment with raltegravir 400 mg orally twice daily for 6 months in 16 patients with HAM/TSP induced a downward trend in HTLV-1 proviral load in PBMC and cerebrospinal fluid (CSF). However, no significant differences were observed in either HTLV-1 proviral load or clinical status. However, immunologic studies indicated a decrease in the number of CD4^+^ CD25^+^ T cells, which is the main reservoir of HTLV-1. Interestingly, the culture study of PBMCs at 6 months after the commencement of the treatment revealed that the frequency of HTLV-1 Tax-positive cells in the population of CD4^+^ CD25^+^ T cells was significantly reduced, with a significant decrease in the frequency of IFN-γ-positive cells in this cell population. In addition, a reduction in spontaneous lymphoproliferation in the culture was also observed at this point. These findings suggest that raltegravir not only functions as an integrase inhibitor, but also inhibits HTLV-1 expression. Very recently, the efficacy of another integrase inhibitor, dolutegravir, was also reported in a phase II controlled trial [16]. This trial was an open-label randomized trial of dolutegravir at 50 mg daily versus oral vitamin C at 500 mg daily for 48 weeks in HAM/TSP patients. The administration of dolutegravir, but not vitamin C, induced a significant improvement in neurologic function, including lower extremity spasticity, sensory function, and nocturia. In addition, very interestingly, a significant decrease in HTLV-1 proviral load with an improvement in cytokine expression in the peripheral blood was observed upon the administration of dolutegravir, but not vitamin C. Indeed, although a high HTLV-1 proviral load in HAM/TSP patients is predominantly maintained by increased proliferation of HTLV-1-infected cells [14], it was also reported that active viral replication followed by cell-to-cell transmission of HTLV-1 via a virological synapse is essential to maintaining the HTLV-1 proviral load [17]. Therefore, the decrease in HTLV-1 proviral load under dolutegravir treatment might be caused by the inhibition of the cell-to-cell transmission of HTLV-1 through the decrease in HTLV-1 replication/expression. Indeed, when considered alongside the finding of a downward trend in the HTLV-1 proviral load in PBMC and CSF with a significant decrease in HTLV-1 Tax-positive cells in the population of cultured CD4^+^ CD25^+^ T cells after raltegravir treatment, as mentioned before, both raltegravir and dolutegravir might have the potential to become candidate therapeutic agents against HAM/TSP.

(c) **Histone deacetylase enzyme inhibition**

As acetylated histones are associated with transcriptionally active chromatin, and deacetylated histones with inactive chromatin, chromatin acetylation is regulated by the balance between histone acetyltransferases and histone deacetylases (HDACs) as a form of epigenetic control under physiological conditions. Thus, histone acetylation plays an important role in the regulation of HTLV-1 gene expression [18,19]; in particular, inhibition of HDAC activity leads to histone hyperacetylation followed by increases in HTLV-1 gene expression. The relationship of HTLV-1 proviral load and/or expression with host immune system factors, such as HTLV-1-specific CTLs, is at equilibrium in the peripheral blood [5]. Therefore, if the HTLV-1 proviral load is increased due to up-regulation of HTLV-1 expression (e.g., in cells infected with the latent or silent form), HTLV-1-specific CTLs are more activated, and the number of HTLV-1-infected cells might be reduced in the peripheral blood. A clinical trial involving oral administration of an anti-epileptic drug, valproate (VPA), which is an HDAC inhibitor, was performed in 16 HAM/TSP patients over 3 months [20]. As a result, HTLV-1 proviral loads in the peripheral blood were significantly decreased in all patients following a transient increase. Although the authors did not describe the changes in clinical status in detail, they mentioned that VPA treatment induced a reduction in spasticity in all patients. However, it was reported that VPA decreased the activity of HTLV-1-specific CTLs, leading to a reduction in the efficiency of CTL surveillance, and the decrease in HTLV-1 proviral load was only transient [21,22]. These findings make estimates regarding the efficacy of HDAC inhibitors in the treatment of HAM/TSP patients pessimistic. Recently, the effects of other HDAC inhibitors, including panobinostat and romidepsin—used as therapeutic agents for multiple myeloma and peripheral T cell lymphomas, respectively—on HTLV-1 Tax expression were analyzed in chronically HTLV-1-infected cells or CD4-positive T cells isolated from HTLV-1-infected patients [23]. As a result, it was shown that these compounds enhance HTLV-1 Tax transcription through Tax-target gene transcription, although Tax protein levels were only moderately enhanced. Furthermore, it was revealed that these compounds induce apoptosis in chronically HTLV-1-infected cells as an additive function, besides their “gene activation” function via histone hyperacetylation, followed by increases in HTLV-1 gene expression. These findings suggest that HDAC inhibitors such as panobinostat and romidepsin have the potential to eliminate HTLV-1-infected cells, thus leading to the possibility of developing novel therapeutic regimens against HAM/TSP.

(d) **Monoclonal antibodies**

Molecular-targeted therapies involving monoclonal antibodies have recently been used as part of therapeutic strategies for various diseases.

(i) **Daclizumab (anti-IL-2 receptor chain Ab)**

Although it is well-known that IL-2 and IL-2 receptor α (IL-2Rα) are induced by HTLV-1 Tax transactivation in HTLV-1-infected cells [24,25], IL-2Rα+T cells also serve as a reservoir of HTLV-1. Therefore, humanized anti-Tac antibody (daclizumab)—the humanized form of monoclonal antibody against IL-2Rα—might have the potential to target HTLV-1-infected cells, thus leading to the elimination of HTLV-1-infected cells. This treatment was part of the first trial considering molecular-targeted therapies using monoclonal antibodies for HAM/TSP patients, in which nine patients with HAM/TSP were treated with daclizumab [26]. As a result, immunological studies revealed selective down-regulation of the number of circulating activated T cells expressing the IL-2R receptor and a decrease in spontaneous PBL proliferation ex vivo, as expected. Furthermore, the HTLV-1 proviral load in the peripheral blood was reduced by an average of 52% after this treatment, suggesting that daclizumab treatment has the potential to selectively remove HTLV-1-infected cells expressing IL-2R from the peripheral blood of HAM/TSP patients. However, this treatment induced mild improvements in the motor disability scores of only three HAM/TSP patients.

(ii) **Mogamulizumab (anti-CCR4 Ab)**

Mogamulizumab is a monoclonal antibody which has been approved as a therapeutic agent for patients with relapsed/refractory or untreated CCR4-positive aggressive adult T-cell leukemia/lymphoma (ATL) [27]. Based on the finding that CD4^+^ CCR4^+^ T cells are the main HTLV-1 reservoir [28], PBMCs from HAM/TSP patients were treated with mogamulizumab [29]. As a result, mogamulizumab effectively reduced the HTLV-1 proviral load, leading to down-regulation of both spontaneous lymphocyte proliferation and IFN-γ production. Subsequently, a Phase I/IIa clinical trial involving 21 HAM/TSP patients was performed [30]. After administration of mogamulizumab at an escalated dose, the changes in clinical status, such as HTLV-1 proviral load in PBMCs and CXCL10/neopterin in cerebrospinal fluid, were observed for 85 days in the phase I study and a further 19 cases continued on to the phase IIa study. Administration of this monoclonal antibody induced a significant reduction in the HTLV-1 proviral load in PBMCs and CXCL10/neopterin in cerebrospinal fluid. Clinically, a reduction in spasticity and a decrease in the motor disability score were observed in 79% and 32% of patients, respectively. Following the phase I/IIa study, in an extended long-term study spanning 4 years, administration of mogamulizumab also induced similar clinical effects to those outlined above, including improvements in spasticity, motor disability, and bladder function. Furthermore, the HTLV-1 proviral load in peripheral blood and CSF decreased by 60.7% and 66.3%, respectively, while the concentrations of neopterin and CXCL10 in the CSF decreased by 37.0% and 31.0%, respectively [31]. At present, there are no clinical trial reports regarding the outcomes and efficacy of mogamulizumab over such a long study period (i.e., 4 years) in HAM/TSP patients. Therefore, a series of clinical trials regarding the use of mogamulizumab in HAM/TSP patients might be valuable at this point, even as an open-label study. The efficacy of this monoclonal antibody treatment has also very recently been reported in a multicenter, randomized, double-blind, placebo-controlled phase III study [32]. This study included a 24-week double-blind, placebo-controlled period, a 24-week open-label period, and an extension treatment period. The mogamulizumab and placebo groups comprised 34 and 33 randomly selected patients, respectively. At the end of the double-blind period, the mogamulizumab arm showed a significant decrease in HTLV-1 proviral load and CSF neopterin/CXCL10 levels. However, no statistically significant clinical benefits were found in the mogamulizumab group. This is a limitation of this study. Although the reason for this is not clear, it might be due to the relatively small sample size and short 24-week double-blind treatment period. Indeed, as the clinical course of HAM/TSP is mostly slowly progressive, an estimation of change in clinical status over a longer period might be necessary. However, when considering both the maintenance of clinical status with HTLV-1 proviral load reduction throughout the open-label and extension treatment periods in this study, and the outcomes of the long-term study spanning 4 years mentioned above, we can conclude that treatment with mogamulizumab may have a promising effect on HAM/TSP patients. However, it is invariably associated with serious adverse effects, such as skin rash and lymphopenia. Therefore, further research must be pursued carefully.

(iii) **Rituximab (anti-CD20 Ab)**

A unique therapeutic regimen has very recently been reported. Single-cell RNA sequencing (scRNA-seq) analysis of PBMCs from HAM/TSP patients indicated a significant effect of HTLV-1-associated B cells on T cells. In addition, HTLV-1 was found to infect B cells, and depletion of B cells inhibited the proliferation of T cells, suggesting that peripheral blood B cells in HAM/TSP patients are, to some extent, involved in the pathogenesis of HAM/TSP [33]. Rituximab is a monoclonal antibody which has been approved as a therapeutic agent for patients with optic myelitis spectrum disorders, among other conditions [34]. Based on these data, 14 patients with HAM/TSP received rituximab treatment twice every 24 weeks and underwent a 48-week follow up [33]. As a result, this treatment was found to induce some clinical benefits, including an improvement in pyramidal function scores with bladder functions, concomitant with a tendency to decrease HTLV-1 proviral loads and infected T cell counts. The outcomes of this study might be of interest, considering the involvement of B cells in the pathogenesis of HAM/TSP.

(e) **Prosultiamine**

Prosultiamine is a homolog of allithiamine, which was originally synthesized from thiol-type vitamin B1 and allicin [35]. Based on the finding that prosultiamine can induce the caspase-dependent apoptosis of HTLV-1-infected cells through disruption of intracellular redox reactions due to the presence of a disulfide moiety in its structure [36], it was administered via the oral route for 12 weeks in 24 patients with HAM/TSP in a single-center, open-label trial [37]. As a result, it was reported that prosultiamine could induce (i) improved motor function in the lower extremities, based on a decrease in spasticity; (ii) amelioration of urinary disturbances; and (iii) a decrease (approximately 15.4%) in the level of HTLV-I provirus in peripheral blood. Of these, the effect on urinary disturbances was the most remarkable outcome, as evidenced by an improvement in detrusor overactivity in a urodynamic study.

(f) **Heparinoid**

(i) **Fucoidan**

Based on the finding that fucoidan—a complex sulphated polysaccharide derived from marine seaweed which is classified as a heparinoid—exerts inhibitory effects on the cell-to-cell spread of HTLV-1 in vitro, fucoidan was administered to 13 patients with HAM/TSP for 6–13 months in a single-center, open-label trial [38]. As a result, this treatment induced a 42.4% decrease in the HTLV-1 proviral load in peripheral blood. Clinically, although a significant improvement was not induced, no exacerbation was observed.

(ii) **Pentosan**

Heparin treatment has been previously reported to be effective in HAM/TSP patients [39]. Pentosan polysulfate sodium (PPS)—another heparinoid—has been safely used in Europe for the past 50 years as a thrombosis prophylaxis and for the treatment of phlebitis. A clinical trial involving the administration of PPS in 12 HAM/TSP patients was conducted with an open-label design [40]. Administration of PPS eight times, once a week, via the subcutaneous route induced marked improvement in lower extremity motor function based on reduced spasticity, leading to a significant increase in soluble vascular cell adhesion molecule (sVCAM)-1 in sera. On the other hand, although the decrease in the HTLV-1 proviral load did not reach statistical significance in this study, a trend of decreased HTLV-1 proviral load was observed in nine patients. Indeed, polysulfates such as PPS and fucoidan have the potential to inhibit the intercellular spread of HTLV-1 by blocking binding of the virus to heparan sulfate proteoglycans through its function as a polyanion [41]. Therefore, PPS treatment might induce a decrease in HTLV-1-infected cells in the peripheral blood in some HAM/TSP patients through the inhibition of the cell-to-cell transmission of HTLV-1. However, it is highly likely that the improved motor function in the lower extremities of HAM/TSP patients is induced by other mechanisms. It has been reported that PPS has the potential to suppress the transmigration of HTLV-1-infected cells, as well as HTLV-1 replication and transmission [42]. At this point, PPS might induce neurological improvement through the inhibition of chronic inflammation by suppressing the transmigration of HTLV-1-infected cells to the spinal cord through blockage of the adhesion cascade by increasing serum sVCAM-1, in addition to promoting rheological improvement in the microcirculation.

(2) **Therapies focusing on immunomodulatory (mainly anti-inflammatory) effects** (Table 1)

Numerous immunomodulatory therapies for the suppression of the chronic inflammatory cascade induced by HTLV-1, which is a powerful causative agent of inflammation, have been assessed in HAM/TSP patients (Table 1). This strategy is mainly focused on anti-inflammatory effects (e.g., the suppression of immune activation, particularly for activated HTLV-1-infected cells), in order to achieve a reduction in chronic inflammation in the spinal cord, through down-regulation of inflammatory cytokines and/or adhesion molecule expression, among others. The regimens also exert effects on activated HTLV-1-non-infected cells, which are subsequently induced by the activation of HTLV-1-infected cells. This section describes some representatives of the therapeutic regimens introduced in Table 1. Discussions of other therapeutic regimens can be found in references [5,43].

(a) **Corticosteroids**

Corticosteroid hormones, such as prednisolone, are the most popular agents for the treatment of HAM/TSP patients at present; however, their efficacy remains controversial. Recently, the efficacy of prednisolone treatment at a mean dose of 4.8 mg/day was reported in a multicenter retrospective cohort study [44]. Subsequently, a randomized controlled trial with corticosteroid treatment was performed (HAMLET-P) [45]. This is the first trial involving a case–control study focused on corticosteroid treatment. The trial was performed in two groups: 8 patients with rapid progressors and 30 patients with slow progressors. For the former, a 3-day course of intravenous methylprednisolone followed by oral prednisolone was administered; for the latter, oral prednisolone or a placebo was administered. An improvement of more than either 1 grade in the Osame Motor Disability Score or 30% in the 10 m walking time at 2 weeks after the commencement of treatment and changes from baseline in the 10 m walking time at week 24 after the commencement of treatment were determined as the primary outcomes for the rapid progressors and slow progressors, respectively. In the rapid progressor group, all four patients with intravenous methylprednisolone and only one of four without intravenous methylprednisolone achieved the primary outcome. In the slow progressor group, the median changes in 10 m walking time were greater in the prednisolone group than in the placebo group. Although there were no statistically significant differences in the primary endpoints between both groups, the overall data seem to indicate the potential benefits of corticosteroid therapy. At present, these findings serve as evidence that corticosteroid treatments, such as the use of both high-dose pulsed methyl prednisolone for induction and low-dose (5 mg) oral prednisolone as maintenance therapy for progressive disease, may provide benefits in HAM/TSP treatment [46]. However, whether or not corticosteroid therapies are effective in the long term and their potential for lifelong treatment remain controversial.

(b) **Interferon-α and -β**

Interferon (IFN)-α and -β, which are type I IFNs, play important roles in multiple biological actions, including anti-viral effects, growth regulation, and modulation of the cellular immune response [47]. Therefore, treatment with IFNs seems to be reasonable for HAM/TSP, as these regimens can target immunological dysregulation due to high HTLV-1 proviral loads in the peripheral blood. IFN-α has proven to be effective in the treatment for HAM/TSP patients in a multicenter, randomized, double-blind, controlled trial [48] and has been approved as a therapeutic agent for HAM/TSP by the Ministry of Health, Labour and Welfare in Japan. Indeed, IFN-α treatment induced a decrease in the HTLV-1 proviral load in the peripheral blood, along with a reduction in spontaneous peripheral blood lymphocyte proliferation and correction of Th1/Th2 imbalance [49,50,51], which deviates toward Th1 in HAM/TSP [52], thus leading to clinical improvement. The efficacy of treatment with IFN-β1a, which has previously been approved for use in the treatment of multiple sclerosis [53], has also been reported for HAM/TSP [54]. Thus, these IFNs seem to have considerable benefits in therapeutic strategies for HAM/TSP. However, whether or not IFN treatments are well-tolerated over the long term and their potential for use in lifelong treatment remain uncertain.

(c) **Hu-Mikβ1(anti-IL-2/IL-15 receptor β chain Ab)**

Research has shown that IL-15 may be involved in the pathogenesis of HAM/TSP [55]. Based on this, Hu-Mikβ1, which is a humanized monoclonal antibody directed toward the IL-2/IL-15 receptor β-chain, was administered intravenously for nine HAM/TSP patients [56]. The inhibition of aberrant CD8^+^ T cell function, including spontaneous lymphoproliferation and degranulation and IFN-γ expression, was induced without significant toxicity, although no clinical efficacy was observed.

(d) **Teriflunomide**

Teriflunomide—which inhibits the mitochondrial enzyme dihydro-orotate dehydrogenase, leading to the inhibition of de novo pyrimidine synthesis—has inhibitory activity against the proliferation of activated T and B cells [57,58]. This drug functions as an immunomodulator and is thus used in the treatment of patients with multiple sclerosis (MS) and rheumatoid arthritis [58,59]. Teriflunomide has been approved by both the US Food and Drug Administration and the European Medicines Agency as a disease-modifying drug for relapsing–remitting MS. In vitro treatment of PBMCs from HAM/TSP patients with this drug induced a significant reduction in HTLV-1 proviral load due to inhibition of the proliferation of CD4^+^ T cells [60]. The efficacy of teriflunomide treatment for HAM/TSP patients has been reported in a very recent triple-blind, randomized, placebo-controlled trial [61]. Administration of teriflunomide (14 mg daily) for 12 months induced clinical amelioration in 11 HAM/TSP patients, including a significant decrease in the OMDS concomitant with a decrease in walking time and improvement in the total score in a urodynamic study, with no serious adverse effects except tolerable liver dysfunction reported in this study. In particular, the HTLV-1 proviral load significantly decreased. As the increased proliferation of HTLV-1-infected cells is predominantly involved in the maintenance of high HTLV-1 proviral load in the peripheral blood of HAM/TSP patients [14], as mentioned above, teriflunomide might be a reasonable drug for achieving a reduction in the HTLV-1 proviral load concomitant with corrective activity regarding immunological activation in the peripheral blood of HAM/TSP patients. In this context, teriflunomide might have the potential to become part of therapeutic regimens for this condition. Therefore, further studies involving clinical trials of longer duration and including a large number of HAM/TSP patients are needed with great vigilance toward hepatotoxicity.

(e) **Danazol**

The efficacy of danazol, which is a derivative of testosterone, as a treatment for HAM/TSP patients has been reported in a placebo-controlled trial [62]. There was a significant improvement in clinical status, including motor disability grading, gait disturbance, pain, and urinary disturbance, in 38 HAM/TSP patients treated with danazol (the initial dose of 200 mg/day was increased to 400 mg/day over 2 weeks) for 6 months compared to 33 HAM/TSP patients treated with a placebo, without serious adverse effects.

(f) **L-Arginine**

An open-label, phase II study with L-arginine—which is used as part of regimens for mitochondrial myopathy, encephalopathy, lactic acidosis, and stroke-like episodes (MELAS) [63]—involving 20 HAM/TSP patients has been performed [64]. The daily administration of L-arginine for 1 week induced clinical improvements such as decreases in walking time in a 10 m walk test and decreases in the overactive bladder symptom score, along with a decrease in the CSF neopterin concentration. Although this effect might be dependent on the activity of L-arginine, which can regulate inflammatory and neuroprotective reactions [65], further research is needed for confirmation.

(3) **Pharmaceuticals with the potential to become therapeutic agents**

Several pharmaceuticals which are available as part of regimens for other conditions have recently been analyzed for their immunological and virological effects in HAM/TSP patients. Although these studies were performed only in vitro, intriguing data were reported. In this section, these pharmaceuticals, which might facilitate the development of novel therapeutic approaches, are introduced.

(a) **ABL1 tyrosine kinase inhibitors**

ABL1 tyrosine kinase is an important enzyme for the survival of chronic myelogenous leukemia (CML) cells [66]. Pathway analysis following microarray analysis of CD4^+^ T cells from HAM/TSP patients revealed increased expression of the ABL1 tyrosine kinase mRNA, suggesting that ABL1 tyrosine kinase is also an important enzyme for the survival of HTLV-1-infected cells in HAM/TSP patients [67]. ABL1 tyrosine kinase inhibitors—such as imatinib, nilotinib, or dasatinib—are available as therapeutic agents for CML [68], and in vitro treatment of CD4^+^ T cells from HAM/TSP patients with these inhibitors induced significant reductions in HTLV-1 proviral loads. In addition, ABL1 siRNA transfection reduced cell viability in HTLV-1-infected cell lines. These data suggest that ABL1 tyrosine kinase may be a key molecular target for HTLV-1-infected cells in the context of HAM/TSP treatment.

(b) **EZH1/2 dual inhibitors**

EZH1 and EZH2, which are the enzyme subunits of the polycomb repressive complex, suppress the gene expression involved in the tumor suppressor through histone H3 lysine 27 trimethylation (H3K27me3) [69]. Based on the finding that EZH2 is overexpressed in ATL and Tax-expressing cells [70], the effects of EZH1/2 dual inhibitors for these cells have been analyzed [71]. It was discovered that the EZH1/2 dual inhibitors effectively depleted HTLV-1-infected cells. Microarray analysis of CD4^+^ CCR4^+^ T cells in the peripheral blood of HAM/TSP patients revealed that the expression of EZH2 mRNA was elevated in this cell population [72]. Based on this finding, the effects of EZH1/2 dual inhibitors on these cell populations were investigated in vitro. It was discovered that EZH1/2 inhibitors significantly inhibited spontaneous proliferation of PBMCs from HAM/TSP patients, with reductions in the frequencies of Ki67^+^ CD4^+^ T cells and Ki67^+^ CD8^+^ T cells. Furthermore, this treatment reduced HTLV-1 proviral loads. In addition, these agents also caused an increase in early apoptosis in HTLV-1-infected cells. Overall, these data suggest that EZH1/2 inhibitors suppress HTLV-1-infected cell proliferation through apoptosis. Thus, EZH1/2 inhibitors, which are already available for the treatment of adult T-cell leukemia/lymphoma patients, may also be effective in treating HAM.

(c) **Unasnemab**

Repulsive guidance molecule-a (RGMa), which is a member of the GPI-anchored glycoprotein family, plays a variety of roles in the development and pathologies of the central nervous system, such as regulation of axonal guidance, differentiation of neuronal stem cells into neurons, and the pathogenesis of CNS diseases including multiple sclerosis, neuromyelitis optica spectrum disorder, cerebral infarction, and spinal cord injury [73]. Interestingly, it has been reported that RGMa inhibits axonal growth after CNS injury [74]. On the other hand, treatment with an anti-RGMa-specific antibody induced the promotion of axonal regeneration and motor recovery after spinal cord injury [73]. A gene expression analysis in CD4^+^ T cells from HAM/TSP patients via DNA microarray revealed significantly increased expression of RGMa mRNA, leading to RGMa protein expression in particular, in CD4^+^ CCR4^+^ cells after 2 days of culture; this was mediated by the activation of regulatory elements by HTLV-1 Tax in conjunction with Sp1 [75]. Although HTLV-1-infected cells (e.g., Tax-inducing JPX9 cells and PBMCs from HAM/TSP patients) induced neuronal damage, the neutralizing antibody against RGMa, unasnemab, alleviated this damage, suggesting that RGMa is involved in neuronal damage in the CNS of HAM/TSP patients. These findings are interesting, allowing for consideration of the pathomechanism of HAM/TSP from another perspective.

(d) **Dimethyl fumarate**

Dimethyl fumarate (DMF) is a drug for relapsing–remitting multiple sclerosis which has been approved by the US Food and Drug Administration and the European Medicines Agency [76]. It has recently been reported that DMF can inhibit proliferation and induce apoptosis in HTLV-1-infected and transformed T cells through suppression of the NF-kB and STAT3 signaling pathways [77]. Furthermore, the effects of DMF on PBMCs from HAM/TSP patients have been analyzed very recently [78]. As a result, in vitro treatment with DMF induced significant inhibition of spontaneous proliferation of CD8^+^, CD4^+^, and HTLV-1-infected cells, along with down-regulated production of inflammatory cytokines such as interleukin-6, tumor necrosis factor-alpha, and interferon-gamma.

All of the pharmaceuticals mentioned above (except for unasnemab) are already approved for use in another medical field. Furthermore, unasnemab is also available for human use. Therefore, further evaluations regarding the efficacy and safety of these pharmaceuticals should be performed, especially clinical trials involving HAM/TSP patients, as soon as possible. Although it is unclear whether or not certain agents—such as ABL1 tyrosine kinase inhibitors and EZH1/2 dual inhibitors, which are presently used as anti-cancer drugs—are tolerable as part of long-term treatments for HAM/TSP patients, four pharmaceuticals introduced in this section might have potential as candidates for use in therapeutic regimens for HAM/TSP patients.

(4) **Key mechanisms for decreasing HTLV-1-infected cell loads**

As mentioned above, when considering the ideal therapeutic strategy for HAM/TSP, the most critical goal is the complete deletion of HTLV-1-infected cells from the peripheral blood in HAM/TSP patients. For example, such strategies might involve (1) targeting of HTLV-1-infected cells using monoclonal antibodies; (2) death (including apoptosis) of HTLV-1-infected cells; (3) inhibition of HTLV-1-infected cell proliferation; (4) inhibition of HTLV-1 replication/expression; (5) inhibition of cell-to-cell transmission of HTLV-1; and/or (6) destruction of HTLV-1-infected cells through increased activity of HTLV-1-specific cytotoxic T cells. The pharmaceuticals with the ability to induce decreases in HTLV-1-infected cells, as reported in this review, are listed in correspondence to each outcome described above in Table 2.

## 2. Conclusions and Perspectives for Future Research

In this review, we summarized representative therapeutic approaches for HAM/TSP from the past to the present, considering the effects and characteristics of each approach. Based on the recognition that HAM/TSP is a chronic inflammatory disease in the spinal cord triggered by HTLV-1 infection, numerous approaches from the point of view of both anti-viral and anti-inflammatory effects have been developed to date. Unfortunately, an optimal therapeutic strategy for HAM/TSP is still not available.

Although the most critical goal is the complete deletion of HTLV-1-infected cells from the peripheral blood in treatment for HAM/TSP patients, there are, unfortunately, no such strategies at present. In this review, we introduced several agents with the potential to enable the development of novel therapeutic regimens. These pharmaceuticals have the ability to induce a decrease in HTLV-1-infected cells as mentioned in Section 1 (4). Therefore, they might play a certain role in achieving this critical goal. In addition, these pharmaceuticals, including those discussed in Section 1 (3), are already approved for use in other medical fields. As the clinical course of HAM/TSP is definitively progressive, leading to deterioration in the quality of life of patients once the myelopathy develops, novel and safe therapeutic regimens which can be commenced as soon as possible following diagnosis (i.e., in the early stage) are urgently needed. Therefore, pharmaceuticals which are already approved for use in other medical fields should be proactively assessed in clinical trials, including case–control studies involving HAM/TSP patients, with careful pharmacovigilance for the potential development of ATL or opportunistic infectious complications.

Simultaneously, the development of new pharmaceuticals which are specialized for HAM/TSP is expected to profoundly impact the lives of those affected by this condition.

## Figures and Tables

**Table 1:** Therapeutic trials for HAM/TSP.

(1) Therapies focusing on anti-HTLV-1 effects
1. Reverse transcriptase inhibition
▪ Zidovudine
▪ Lamivudine
▪ Zidovudine + Lamivudine
2. Integrase inhibition
▪ Raltegravir
▪ Dolutegravir
3. Histone deacetylase enzyme inhibition
▪ Valproate
4. Monoclonal antibodies
▪ Daclizumab (anti-IL-2 receptor α chain Ab)
▪ Mogamulizumab (anti-CCR4 Ab)
▪ Rituximab (anti-CD20 Ab)
5. Prosultiamine
6. Heparinoid
▪ Fucoidan
▪ Pentosan
(2) Therapies focusing on immunomodulatory effects
1. Corticosteroid
▪ Oral prednisolone
▪ Intravenous methylprednisolone
2. Interferon-α and -β
3. Plasmapheresis
4. Ciclosporin A
5. Pentoxifylline
6. High-dose intravenous gamma globulin
7. Intermittent high-dose Vitamin C
8. Erythromycin
9. Fosfomycin
10. Fermented milk drink
11. Hu-Mikβ1(anti-IL-2/IL-15 receptor β chain Ab)
12. Teriflunomide
13. Danazol
14. L-Arginine

**Table 2:** Key mechanisms for decreasing HTLV-1-infected cell loads and corresponding pharmaceutical agents.

(1) Targeting of HTLV-1-infected cells by monoclonal antibodies:
daclizumab, mogamulizumab, rituximab
(2) Death (including apoptosis) of HTLV-1-infected cells:
prosultiamine, panobinostat/romidepsin, ABL1 tyrosine kinase inhibitors, EZH1/2 dual inhibitors, dimethyl fumarate
(3) Inhibition of HTLV-1-infected cell proliferation:
teriflunomide, EZH1/2 dual inhibitors, dimethyl fumarate
(4) Inhibition of HTLV-1 replication/expression:
integrase inhibitors (raltegravir, dolutegravir), pentosan
(5) Inhibition of cell-to-cell transmission of HTLV-1:
fucoidan, pentosan
(6) Destruction of HTLV-1-infected cells through increased activity of HTLV-1-specific cytotoxic T cells:
HDAC inhibitors (valproate, panobinostat/romidepsin)

## Data Availability

The original contributions presented in this study are included in the article. Further inquiries can be directed to the corresponding author(s).
